# Supplementary figures and images for: Systems analysis of immune responses to attenuated P. falciparum malaria sporozoite vaccination reveals excessive inflammatory signatures correlating with impaired immunity
Source: PLoS Pathog. 2022 Feb 2;18(2):e1010282. doi: 10.1371/journal.ppat.1010282 (PMC8843222; doi:10.1371/journal.ppat.1010282)

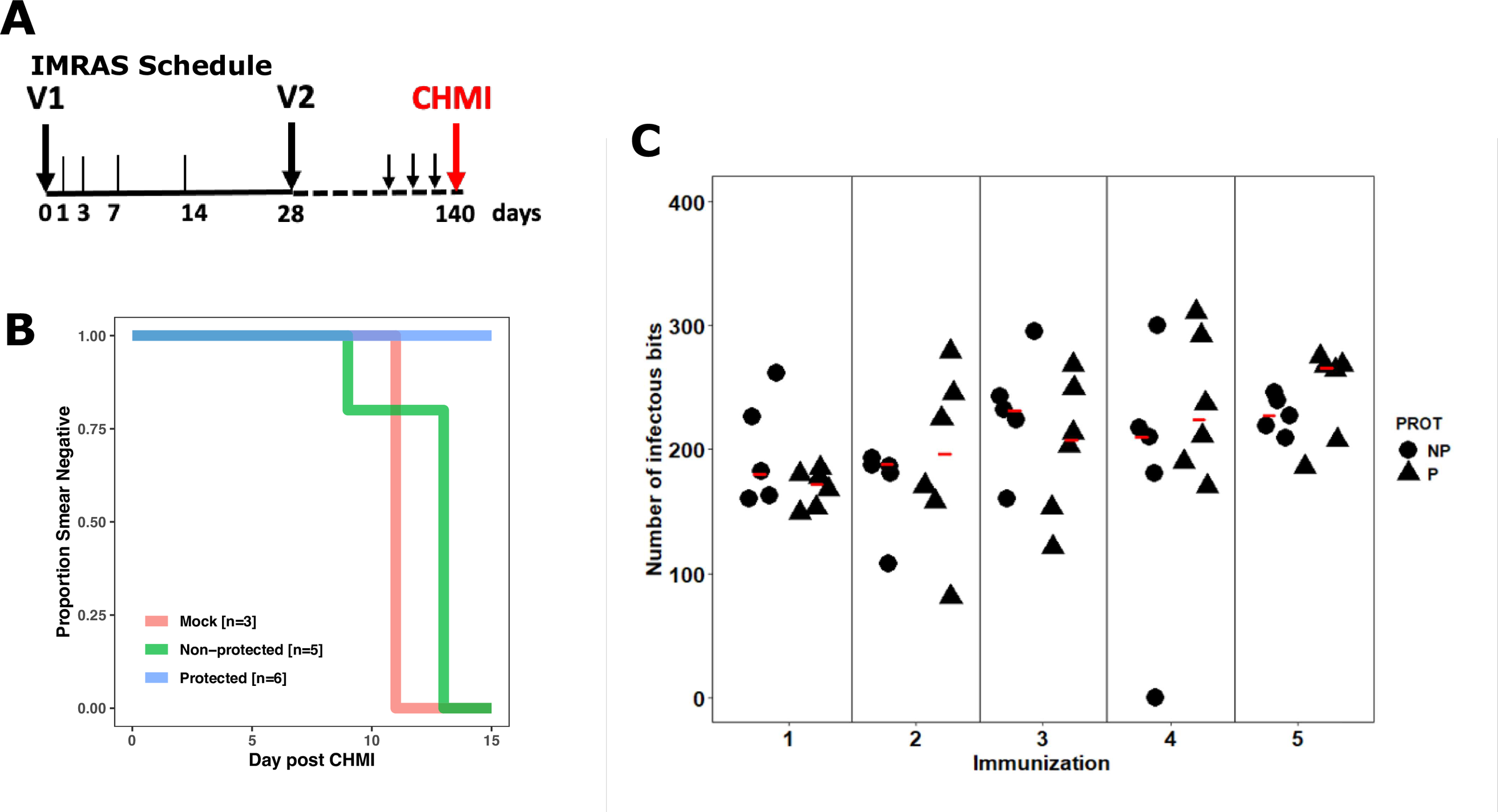

Supplement: S1 Fig — A. Schematic indicating timing of vaccination and sampling. Black vertical arrows indicate immunizations, with the first (V1) and second (V2) immunization indicated. Blood samples taken between V1 and V2 indicated with vertical black lines. Time of CHMI shown as a red vertical arrow. B. Kaplan-Meier curve showing days to thick blood smear positivity for IMRAS subjects who were RAS-immunized (protected/non-protected) or mock immunized. C. Number of infectious mosquito bites received by each subject at each immunization. Circles and triangles indicate non-protected and protected subjects, respectively. Red lines indicate the median number of infectious mosquito bites. (TIF) [file ppat.1010282.s001.tif]

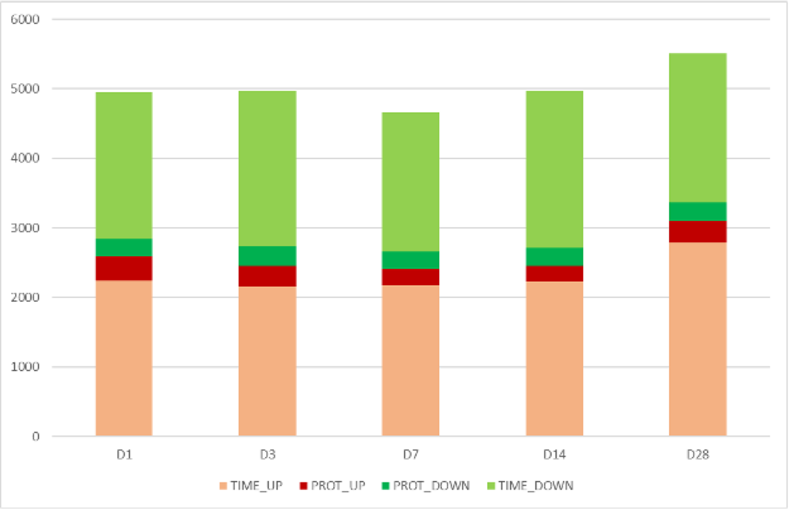

Supplement: S2 Fig — Barplot showing numbers of vaccine-induced genes with increased (pink/red) or decreased (light green/dark green) expression relative to day 0 in all immunized subjects (TIME_UP+PROT_UP, TIME_DOWN+PROT_DOWN) (FDR < 0.2, p <0.05, 90% CI > 0 or < 0). Darker colors indicate genes that additionally differ significantly in expression between protected (P) and non-protected (NP) subjects (PROT_UP, PROT_DOWN). (TIF) [file ppat.1010282.s002.tif]

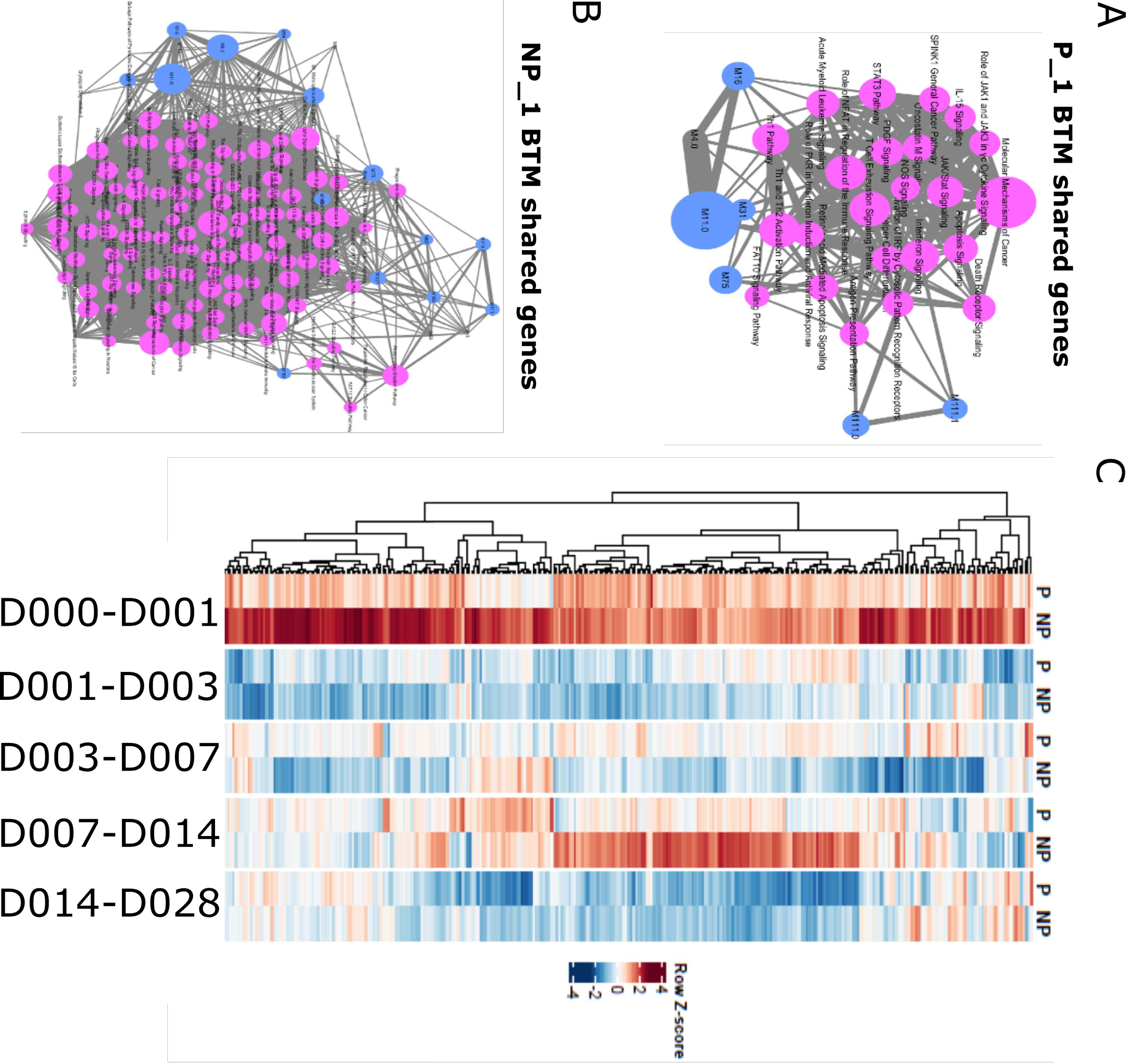

Supplement: S3 Fig — A,B. Gene overlap of IPA pathways and BTMs enriched in A. cluster P_1 and B. NP_1, Node sizes indicate numbers of genes in a BTM or IPA pathway, and line thickness indicates the numbers of shared genes between two nodes. C. Heatmap showing expression of 317 genes common in cluster 1 of P subjects and cluster 1 of NP subjects. Expression values were z-score transformed in rows for visualization. (TIF) [file ppat.1010282.s003.tif]

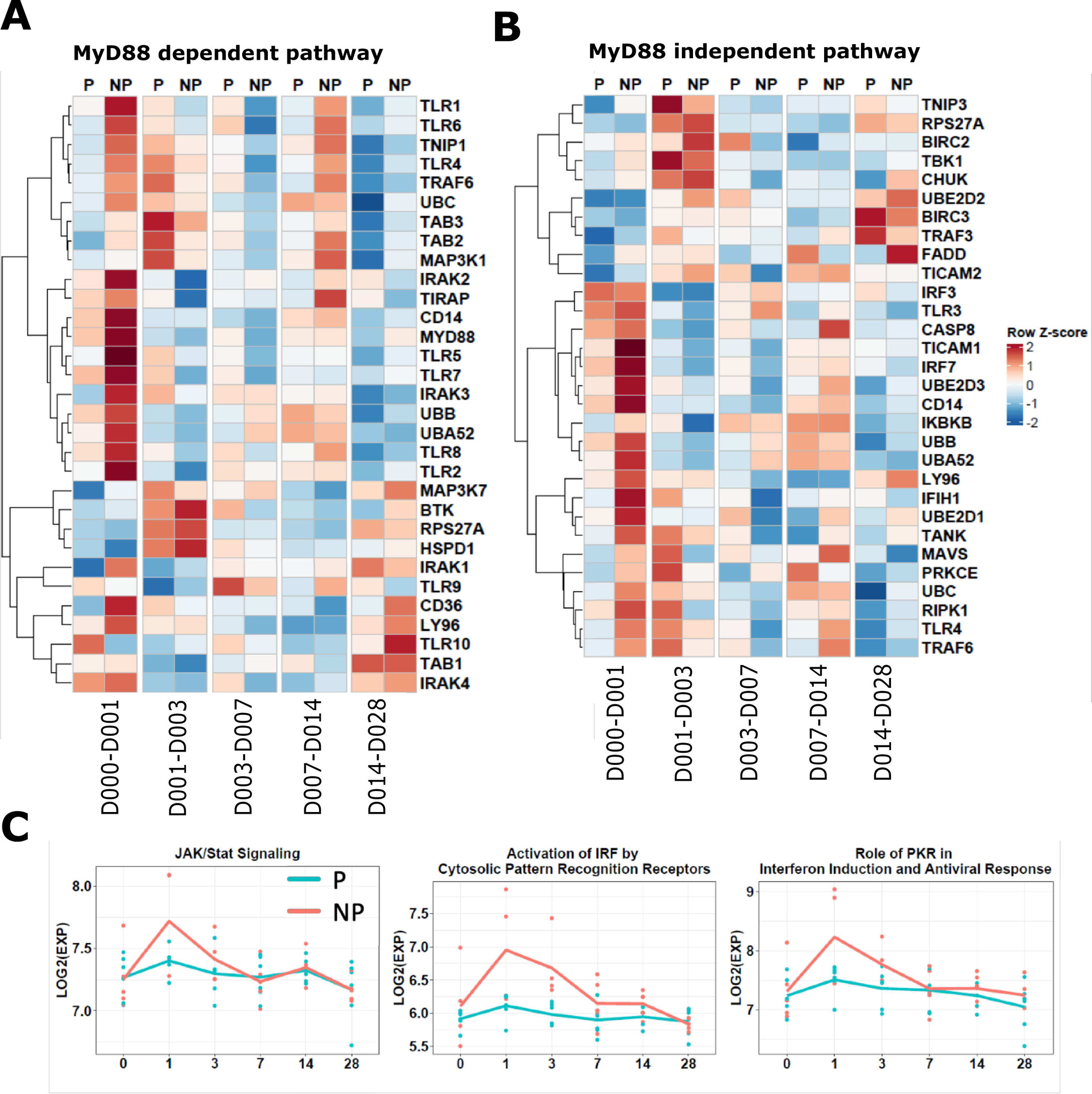

Supplement: S4 Fig — A,B. Heat maps of expression changes for A. MyD88-dependent toll-like receptor associated genes and B. MyD88-independent toll-like receptor associated genes in P and NP subjects. Genes shown were selected using Gene-ontology (GO) annotations GO:0002755 (MyD88 dependent TLR signalling pathway) and GO:0002756 (MyD88 independent TLR signalling pathway). Expression values were Z-score transformed in rows for visualization. C. Average gene expression of selected IPA pathways over time in P (green) and NP (red) subjects. Dots represent average gene expression values per-individuals and solid line represents the average gene expression of the IPA pathway across all participants. (TIF) [file ppat.1010282.s004.tif]

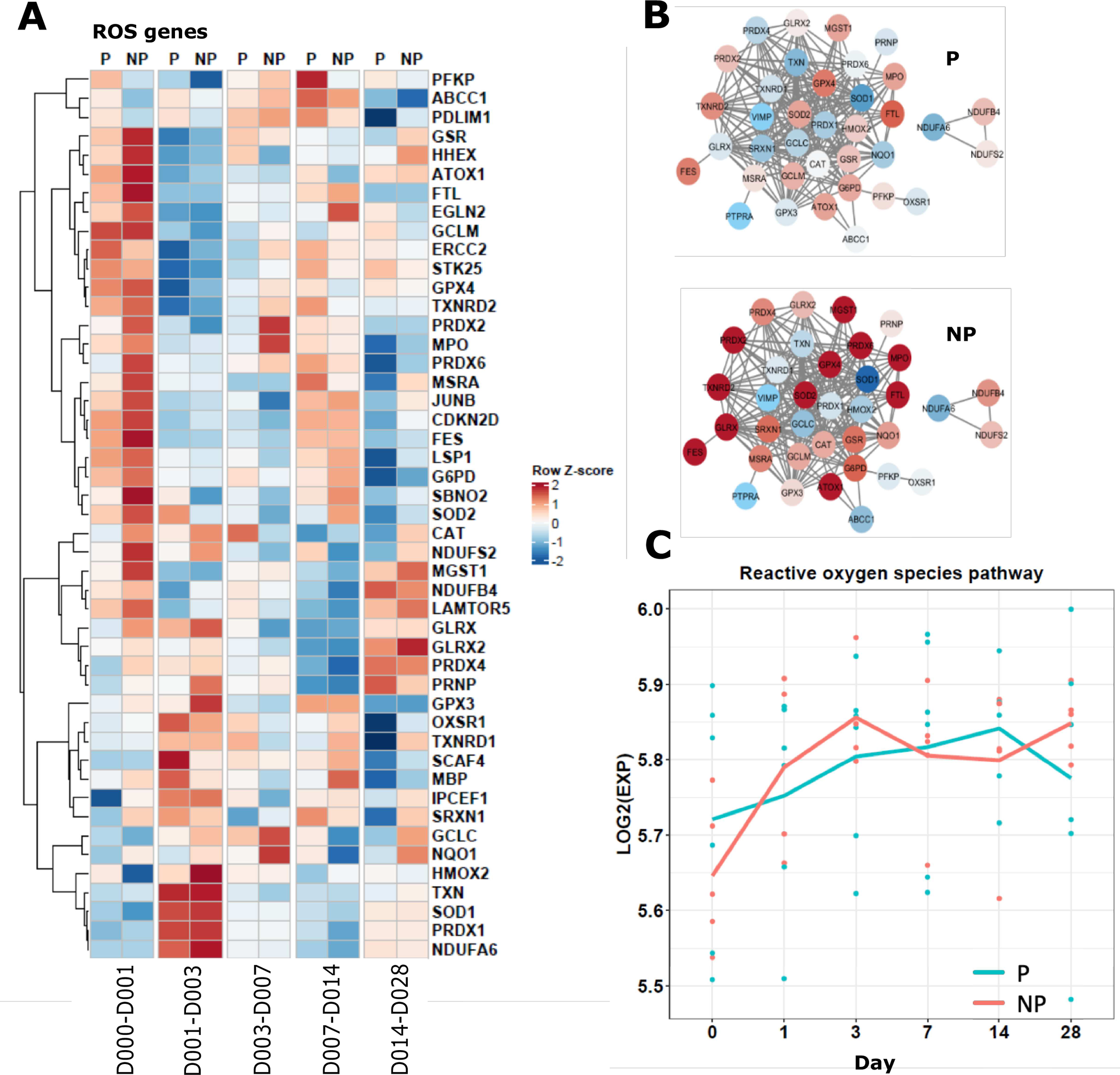

Supplement: S5 Fig — A. Heatmap showing expression of Hallmark Reactive Oxygen Species (ROS) Pathway genes. Expression values were z-score transformed in rows for visualization. B. STRING-DB derived protein-protein interaction networks of ROS genes, colored by expression changes on day 1 compared to day 0 seperately for P and NP. C. Average expression profiles of genes of Hallmark Reactive Oxygen Species Pathway in P and NP subjects. Dots represent average expression in individuals and solid line represents the average expression of the pathway over all P or NP subjects. (TIF) [file ppat.1010282.s005.tif]

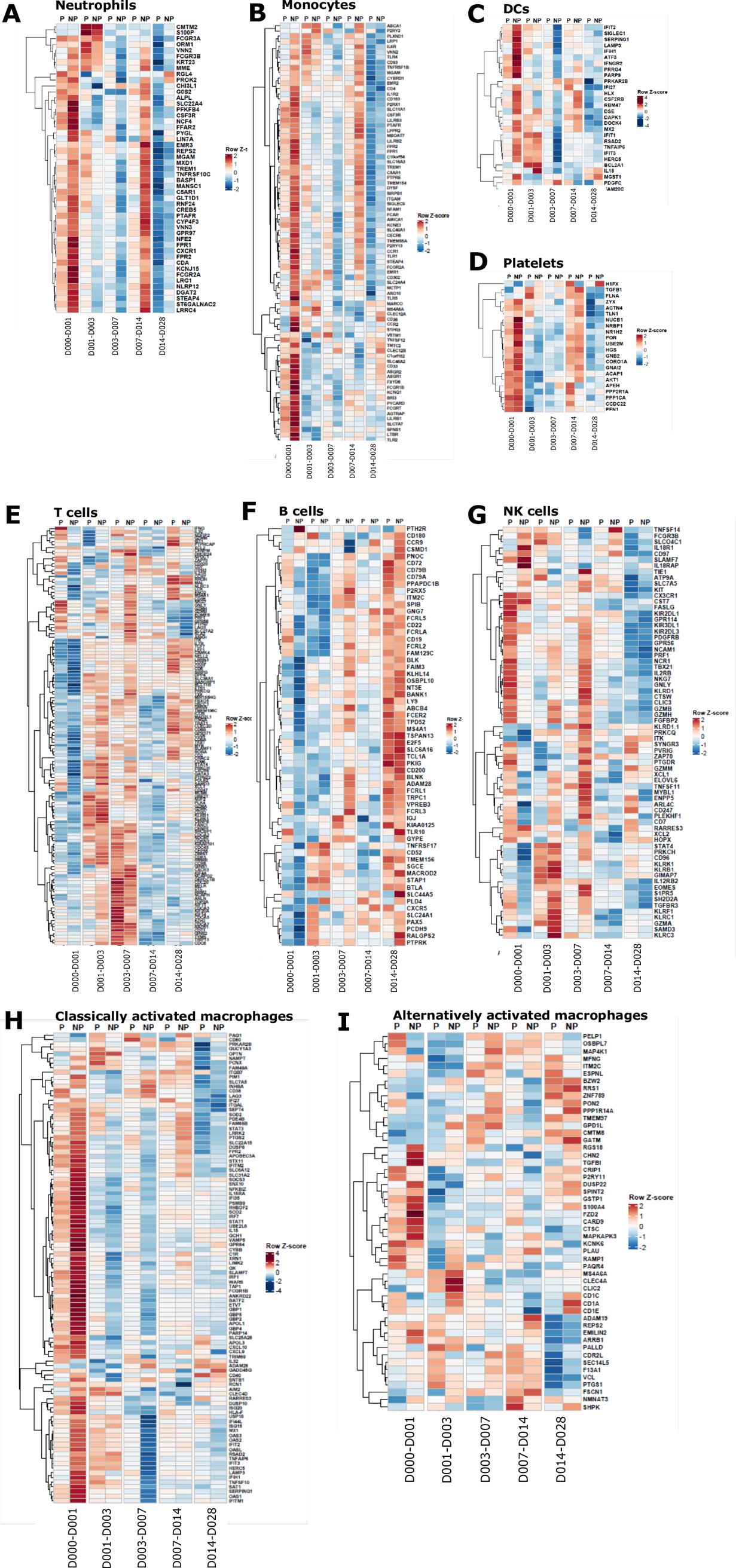

Supplement: S6 Fig — Heatmaps showing expression of genes of cell-type specific pathways: A. Neutrophils, B. Monocytes, C. DCs, D. Platelets, E. T-cells, F. B cells, G. NK cells, H. classically activated macrophages and I. alternatively activated macrophages. Expression values were z-score transformed in rows for visualization. (TIF) [file ppat.1010282.s006.tif]
